# Carrier-Envelope Phase Control in Terahertz Pulse Generation Using InAs Ribbon Metasurfaces

**DOI:** 10.1021/acsphotonics.5c00941

**Published:** 2025-07-28

**Authors:** Sarah Norman, Hyunseung Jung, James Seddon, Samuel Prescott, C. Thomas Harris, Sadhvikas Addamane, Igal Brener, Oleg Mitrofanov

**Affiliations:** † Electronic and Electrical Engineering, 4919 University College London, Torrington Place, London WC1E 7JE, U.K.; ‡ 559754 Center for Integrated Nanotechnologies, Sandia National Laboratories, Albuquerque, New Mexico 87123, United States; § 1105 Sandia National Laboratories, Albuquerque, New Mexico 87123, United States

**Keywords:** terahertz generation, metasurfaces, carrier-envelope phase, shift currents, lateral photocurrents

## Abstract

Generation of broadband terahertz (THz) pulses with variable polarization and carrier-envelope phase can enable the tailoring of THz beam wavefronts for advanced applications in THz imaging and spectroscopy and for strong THz field optics. While metasurfaces composed of deeply subwavelength THz emitters have recently been demonstrated to define the polarization and spatial profile of the generated THz fields, precise phase control or synthesis of THz pulse waveforms remains a challenging problem. Here, we propose and demonstrate metasurfaces composed of indium arsenide (InAs) nanoscale ribbon arrays capable of generating THz pulses with variable carrier-envelope phase. We show that different THz generation mechanisms, each contributing distinct phases, can be activated in the ribbons, enabling carrier-envelope phase control spanning a range of π over a wide band of frequencies (∼1–3 THz). This is achieved solely through the ribbon array geometry using linearly polarized optical excitation of the ribbons. The arrays enable precise control of the THz phase and amplitude, opening the door to advanced structured THz wavefront synthesis using ultrathin dielectric metasurfaces.

## Graphical abstract

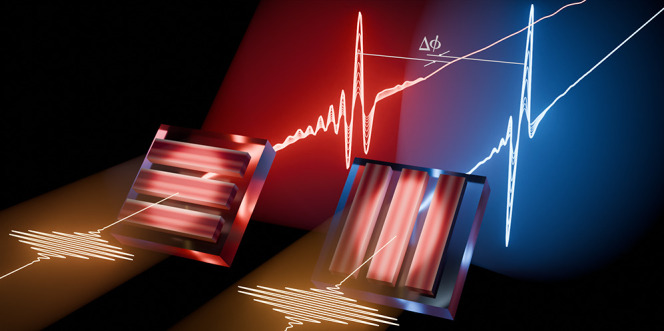

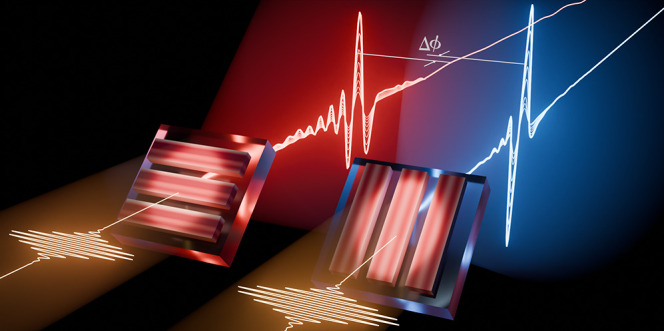

## Introduction

Optical excitation of electronic materials with femtosecond pulses creates a nonequilibrium free charge carrier distribution, driving ultrafast currents that lead to the generation of broadband terahertz (THz) pulses.^1–6^ Nanostructuring of these materials is now used to control a variety of recently discovered underlying mechanisms. They include spin, injection, and shift currents and enable deeply subwavelength THz emitters, which, when integrated into metasurfaces, promise a versatile platform for generating structured THz wavefronts with defined polarization, amplitude, and carrier-envelope phase.^7–23^ Simultaneous THz wave generation and structuring have already produced focused THz beams, Bessel beams, and Airy beams.^24–27^ While nanoscale THz emitters have enabled the control of the THz field vector orientation, including polarity switching (equivalent to a π-phase shift^28–30^ ), the generation of THz pulses with an arbitrary carrier-envelope phase, which could unlock further advances in THz holography, THz scanning tunneling microscopy, and nonlinear THz optics, remains challenging.^31–33^

Indium arsenide (InAs) is one of the most efficient THz emitters,^34–37^ with selected mechanisms predicted to exhibit a phase shift of π/2.^4,38^ Specifically, the THz electric field generated by shift currents (resonant nonlinear process) is expected to follow the rate of optical intensity change, *E*_shift_(*t*) ∝ d*I*(*t*)/d*t*,^4^ while the field from transient photocurrents follows the intensity evolution itself, *E*_lat_(*t*) ∝ ∼*I*(*t*) (for fs optical excitation).^39^ Controlled superposition of these mechanisms could enable the generation of arbitrary THz pulse waveforms and precise manipulation of the THz wave spatial structure; yet, it has not been achieved. More generally, the continuous phase control of THz fields or the synthesis of THz pulses with desired waveforms using any dielectric THz emitter metasurfaces has not been demonstrated. Here, we develop and demonstrate InAs metasurfaces enabling the generation of THz pulses with carrier-envelope phase, ϕ, spanning a range of ±π/2. We achieve this by developing InAs ribbon arrays capable of activating multiple THz generation mechanisms through their size and orientation. In contrast to passive THz metasurfaces,^19,20,31,32,40,41^ the control of phase in THz emitter metasurfaces is achieved over a broad band of frequencies ∼1–3 THz. These emitters could enable precise phase control for tailored phase landscapes within a single ultrathin THz metasurface, unlocking the possibilities for defining not only the field direction and amplitude in the THz beam wavefront on the deeply subwavelength scale, but also the phase, and promising advanced THz spectroscopy and imaging applications.^33,42–44^

## Results and Discussion

To enable THz emitters for the generation of THz pulses with a variable phase using InAs, which supports both the transient photocurrents and resonant nonlinear processes, we selectively activate and control THz generation mechanisms through the geometry of nanoscale InAs ribbons. First, shift currents (arising from an above-bandgap pulsed optical excitation) are maximized in InAs ribbons designed to support optical dipolar resonances and enhance absorption.^11,30^ Second, to enable THz pulse generation with a π/2 phase shift, lateral photocurrents are activated by exploiting the effect of charge carrier density gradient (lateral photo-Dember effect) formed as the excitation pulse sweeps across the surface at oblique angles.^45,46^ The latter mechanism is controlled by the orientation of the ribbons (Figure 1a). Furthermore, we exploit the ribbon’s width to control surface-normal transient photocurrents by altering the resonant field distribution within the ribbons, leading to enhanced THz emission. The combination of these three mechanisms, in principle, provides a means to achieving arbitrary carrier-envelope phase in generated THz pulses.

**Figure 1:**
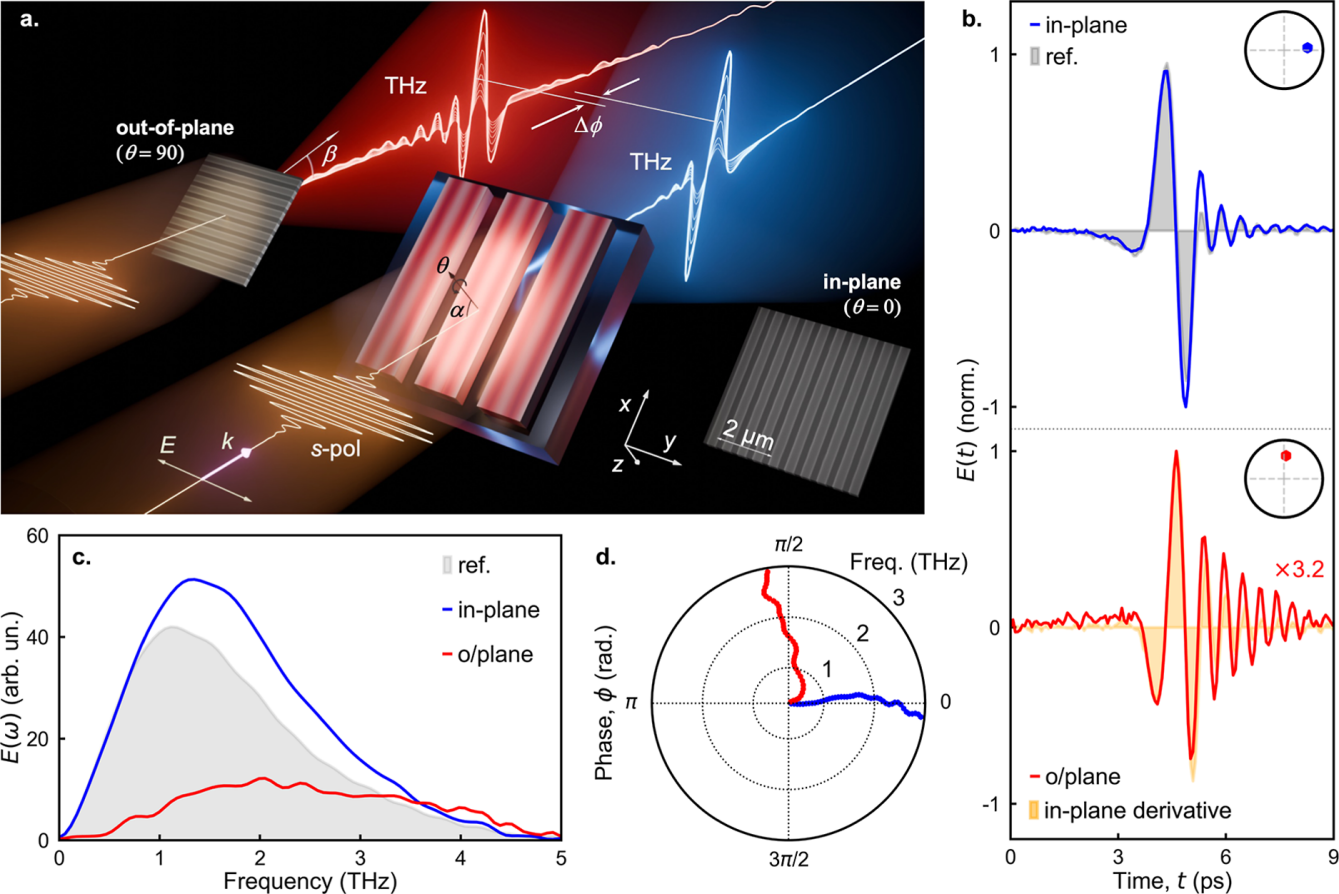
InAs ribbon arrays for THz pulse generation with different carrier-envelope phase Δϕ. (a) Illustration of the THz pulse generation configuration: the InAs ribbon arrays are tilted by angle α = 45° with respect to the optical axis. SEM images show the in-plane (θ = 0°) and out-of-plane (θ = 90°) ribbon arrays and the corresponding generated THz pulses, which exhibit a phase shift Δϕ (blue and red waveforms, respectively). (b) Normalized time-domain waveforms of *p*-polarized THz emission from in-plane (blue) and out-of-plane (red) ribbons when illuminated with *s*-polarized optical excitation. The gray shaded waveform shows the reference pulse generated from a uniform InAs layer (with *p*-polarized incident excitation). The orange-shaded waveform shows the temporal derivative of the pulse generated from the in-plane ribbons. Ribbons have a 400 nm period and 210 nm width. Insets: polar diagrams illustrating the phase of the emitted waves at 1 THz (relative to a uniform InAs layer). (c) Spectral amplitude of the generated pulses for in-plane (blue) and out-of-plane (red) ribbons arrays shown in (b), and (d) phase relative to uniform InAs layer.

We fabricated ribbon arrays from a 130 nm thick layer of (100) InAs and transferred them onto a sapphire substrate (see Supporting Information, Section 1). The arrays were photoexcited with 100 fs, 800 nm, *s*-polarized optical pulses from a Ti:sapphire laser (Figure 1a). The emitted forward-propagating THz pulses were detected using a THz time-domain spectroscopy (THz-TDS) system equipped with a photoconductive antenna (PCA) detector with a small(10 × 10 μm^2^) input aperture positioned ∼6 mm from the ribbon array (see Supporting Information, Section 2).

We observe distinctly different THz pulses for InAs ribbons oriented in the plane of incidence (in-plane) and out of the plane of incidence (o/plane) (Figure 1b–d). The THz pulse waveform generated from the out-of-plane ribbons in Figure 1b matches the temporal derivative of the pulse generated from the in-plane ribbons (red line and orange shaded region in Figure 1b). The corresponding phase difference of ∼π/2 is evident in the polar plot of the Fourier spectra (Figure 1d) and spans over a larger portion of the generated pulse spectra, ∼1–3 THz.

To verify the correlation between the phase shift and ribbon orientation, we incrementally rotate the ribbons around the surface normal (from θ = 0° to θ = 90°; see Figure 1a) and record the resulting THz waveforms (Figure 2a). At θ = 0°, we observe strong THz emission. As the angle increases, the carrier-envelope phase gradually shifts, reaching a value of π/2 at θ = 90°. At the same time, the amplitude of THz emission decreases, reaching a minimum at θ = 45° and then partially recovering at θ = 90°. The amplitude of THz emission from out-of-plane ribbons is ∼3 times weaker than that from in-plane ribbons (Figures 1c and 2a). Since only in-plane ribbons can support lateral photocurrents along the *x*-axis, this suggests that the larger amplitude emission at θ = 0° originates from lateral photocurrents, while the emission at θ = 90° is likely dominated by shift currents, exhibiting a distinct phase shift relative to the emission due to lateral photocurrents. This is consistent with the models for the shift currents and transient photocurrents, and it highlights the role of ribbon orientation in controlling the phase of THz emission.

**Figure 2:**
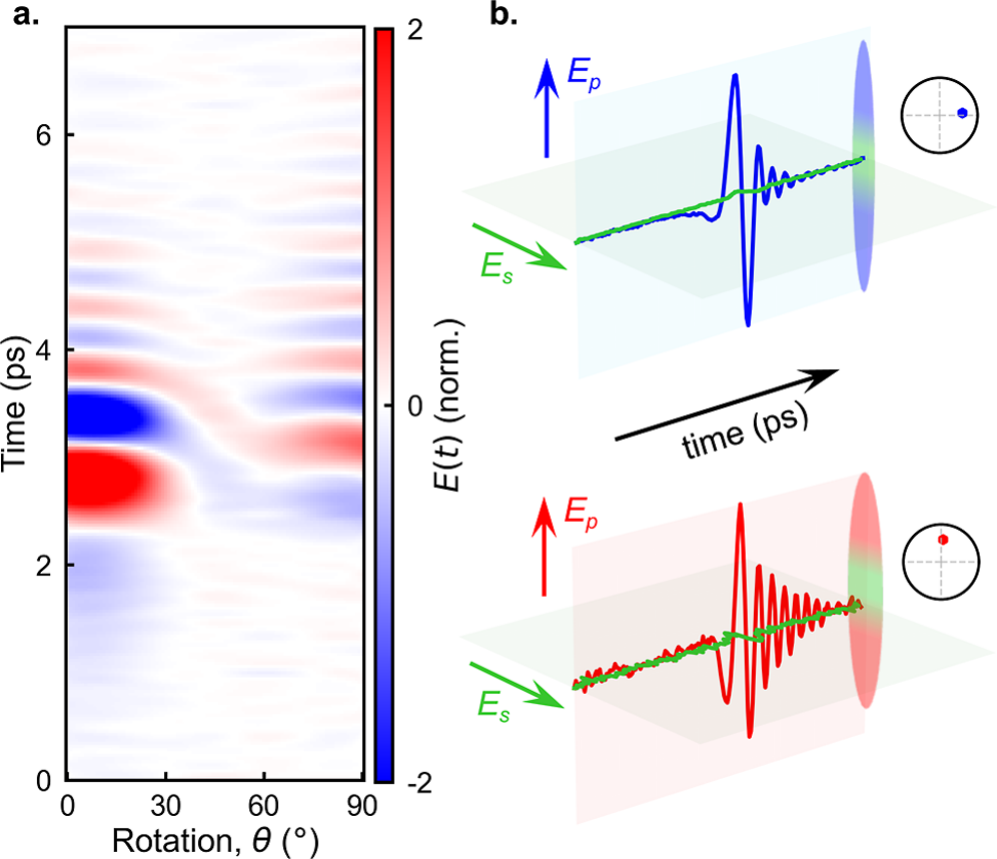
(a) Map of the THz pulse field (time-domain) emitted from an InAs ribbon array (period *P* = 400 nm, ribbon width *W* = 210 nm) showing the variation of THz pulse waveforms as the ribbons are rotated from θ = 0° to θ = 90°. (b) Polarization of the emitted THz pulses: top panel: *p*-polarized (blue) and *s*-polarized (green) components for the in-plane ribbons; bottom panel: *p*-polarized (red) and *s*-polarized (green) components for the out-of-plane ribbons. Insets: polar diagrams illustrating the phase of the emitted waves at 1 THz (relative to a uniform InAs layer).

Before we discuss the details of the emission mechanisms, we first characterize the properties of generated THz pulses for both ribbon orientations. The emitted THz pulses are *p*-polarized, as can be seen in waveforms for both the *p*-polarized and *s*-polarized components of the THz electric field in Figure 2b. To characterize the angular distribution of the emitted waves, we mapped their far-field emission patterns at a distance of ∼6 mm : both samples exhibit relatively symmetric directional emission (Figure 3).

**Figure 3:**
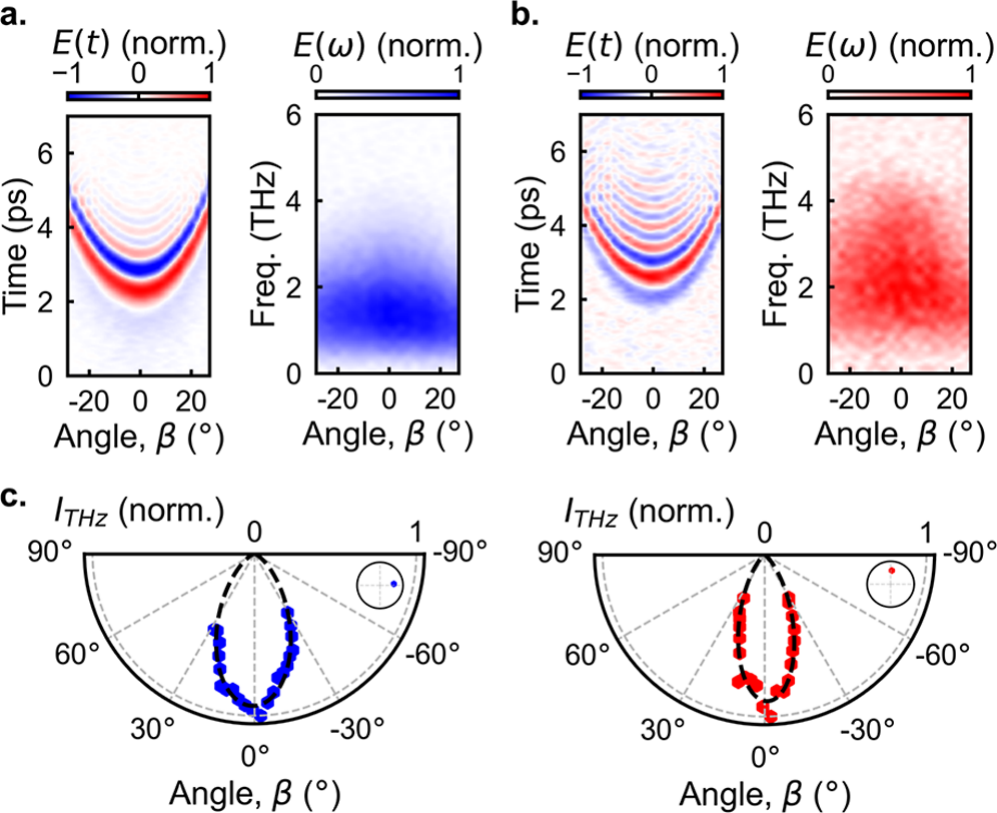
Far-field maps of THz fields and corresponding spectral amplitude maps for THz pulses generated from the in-plane (a) and out-of-plane ribbons (b). (c) Far-field emission patterns (intensity, *I*_THz_) for the in-plane (blue) and out-of-plane (red) ribbons. Insets: polar diagrams illustrating the phase of emitted waves at 1 THz.

For the in-plane ribbons (Figure 3a), the THz emission exhibits a higher amplitude and peaks at lower frequencies than that of out-of-plane ribbons (Figure 3b). The difference in the spectral content is consistent with the anticipated dominant emission mechanisms: the out-of-plane ribbons and the corresponding shift current mechanism are expected to produce THz emission of higher frequencies compared to the transient photocurrents for in-plane ribbons. This difference also translates into a slight variation in the THz emission pattern: the broader spectrum with higher frequency components results in a smaller beam divergence. In contrast, the emission from in-plane ribbons, dominated by lateral photocurrents, peaks at lower frequencies and exhibits a larger divergence (Figure 3c), as expected for sources of spatial extent comparable to the wavelength (see Supporting Information, Section 2).

Interestingly, there is a subtle asymmetry in the emission pattern for in-plane ribbons, showing more pronounced oscillations for the emission angle of β = −20° compared to β = 20° (Figure 3a). This asymmetry can arise from the interferences of two sources of lateral photocurrent: one induced by the excitation pulse sweeping along the ribbons and the other from opposite gradients of the photoexcited charge carrier density for positive and negative angles β (Figure 1a).^45^ Their interference results in a slightly asymmetric far-field emission pattern. By comparison, for out-of-plane ribbons, where neither transient currents contribute to the THz emission, the far-field pattern remains symmetric (Figure 3b).

These results demonstrate that InAs ribbon arrays can enable phase control of emitted THz radiation (Figure 1) without the need to adjust the polarization of the excitation beam.^11^ The emission from these ribbons is linearly polarized (Figure 2) with a symmetric emission pattern defined by the spatial extent of the illuminated array (Figure 3), making them appropriate for use as subwavelength-size point sources of THz emission. By combining the in-plane and out-of-plane ribbon arrays with appropriate weighting to account for the difference in emission amplitude, continuous phase adjustment between 0 and π/2 can be achieved in a single metasurface through geometry alone, enabling structured THz wavefront generation (see Supporting Information, Section 3).

The experimental results suggest the amplitude variation between the in-plane and out-of-plane ribbons and the difference in THz field phase are a result of two different generation mechanisms. In-plane ribbons of various sizes consistently generate THz pulses of larger amplitudes and practically identical phase (Figure 4a; see Supporting Information, Section 4), while out-of-plane ribbons emit a weaker THz field and exhibit either a π/2 or −π/2 phase shift (see Supporting Information, Section 4). This large amplitude and phase shift indicate that the dominant mechanism in the in-plane ribbons is supported only by the ribbons with that orientation. Notably, when we vary the ribbons’ size and period (see Supporting Information, Sections 1 and 4), we observe a clear peak in THz emission amplitude for in-plane ribbons with a period of ∼450 nm (Figure 4a). Below, we will use this size dependence and numerical modeling to identify the relevant mechanisms.

**Figure 4:**
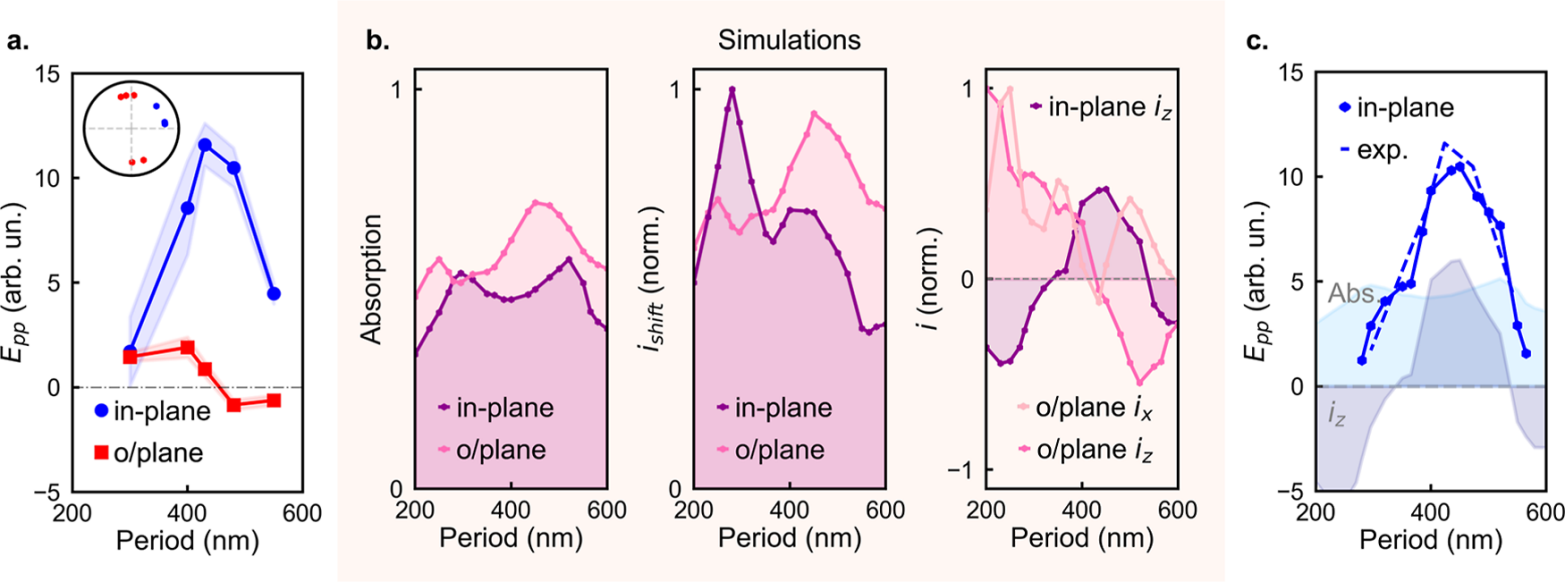
(a) Peak amplitude of THz pulses from in-plane (blue) and out-of-plane (red) ribbon arrays of periods, *P* = 300–550 nm. Inset: polar diagrams showing the phase of emitted waves at 1 THz. (b) Simulated optical absorption at λ = 800 nm (left); simulated THz nonlinear polarization due to shift currents, *i*_shift_ (middle); and simulated charge density gradient photocurrents, *i*_*x*_, *i*_*z*_ (right). (c) Peak THz pulse amplitude from ribbons of periods, *P* = 300–550 nm, compared to the fitted superposition of modeled emission due to the lateral photocurrents and charge density gradient current *i*_*z*_ for the in-plane ribbon arrays.

All THz generation mechanisms scale with the absorption of the pump pulse (a measure of the total number of carriers generated within the ribbon arrays), whereas the nonlinear generation mechanisms, including shift currents, are also modulated by the distribution of vectorial components of the excitation field(see Supporting Information, Sections 5 and 6).^30^ To quantify these contributions, we numerically modeled the total absorption and total nonlinear polarization for the ribbons of both orientations and various sizes. We found that both in-plane and out-of-plane ribbons exhibit comparable levels of absorption (Figure 4b, left) and nonlinear polarization (Figure 4b, middle). Therefore, the drastic increase in THz emission for the in-plane ribbons cannot be explained by either the total number of generated charge carriers or by the field distribution in the ribbons, but rather by the ability of in-plane ribbons to support one mechanism, which is also suppressed for out-of-plane ribbons. The only such mechanism is the lateral photocurrent along the *x*-axis arising due to the gradient of charge carrier density as the pump pulse sweeps along the ribbons.^45,46^

However, the reduced emission at smaller in-plane ribbon sizes suggests the lateral current can still be suppressed or canceled out. While the narrower cross-section in smaller ribbons is more likely to suppress the lateral current due to scattering on surface defects, we also considered whether the currents normal to the surface, *i*_*z*_, could affect the emission (Figure 4b, right). *i*_*z*_ can be excited if the optical intensity distribution in the ribbons varies with depth due to the mode profile. Indeed, our modeling of the optical intensity distribution shows that for ribbon sizes of ∼450 nm, the intensity is higher at the bottom surface of the ribbons, and the corresponding *i*_*z*_ currents (Figure 4b, right) exhibit a peak, adding constructively to the emission from lateral photocurrents. For smaller ribbon sizes, *i*_*z*_ changes sign and destructively interferes with the lateral photocurrent. *i*_*z*_ therefore offers a potential explanation for both the observed peak in emission at *P* = ∼450 nm and the reduced emission at smaller ribbon sizes (Figure 4c). Figure 5 illustrates how the emission due to *i*_*z*_ for in-plane ribbons can interfere destructively (Figure 5a) or constructively (Figure 5b) with the emission due to the lateral photocurrents, while Figure 5c shows how the direction of *i*_*z*_ changes with ribbon size, resulting in the peak in THz emission at *P =* ∼450 nm.

**Figure 5:**
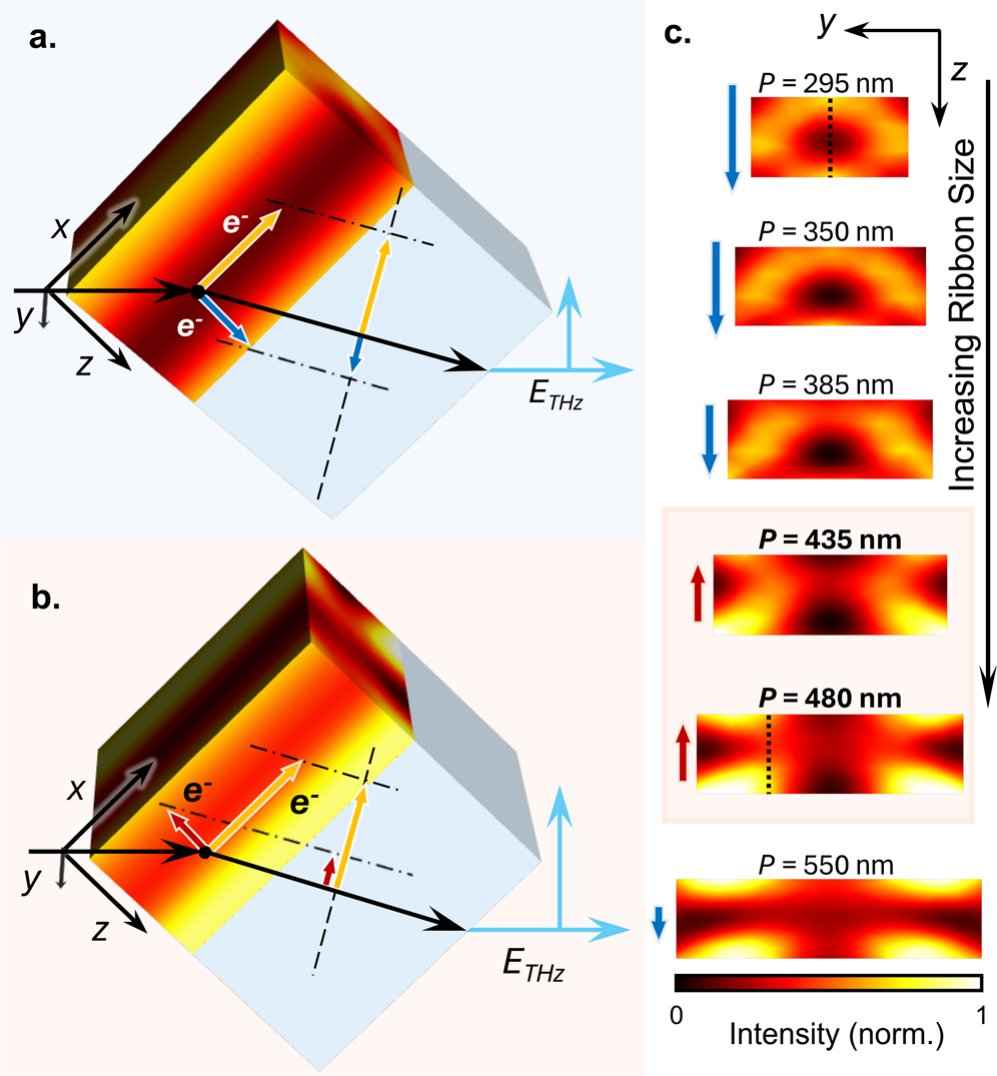
Destructive (a) and constructive (b) interference of the THz field emitted due to the lateral photocurrent (yellow arrows) and charge density gradient current, *i*_*z*_ (blue and red arrows), for the in-plane ribbons (*P* = 295 nm and *P* = 480 nm). (c) Electric field distribution in the *yz*-plane for in-plane ribbons of increasing size (labeled by period *P* in nm). Arrows indicate the magnitude and direction of the total charge density gradient current, *i*_*z*_, and the dotted lines mark the cross sections shown in (a,b), respectively.

For the out-of-plane ribbons, the lateral photocurrent mechanism no longer exists. Furthermore, the *i*_*z*_ current and the in-plane current within the ribbon width, *i*_*x*_, largely cancel out for most ribbon sizes considered here (Figure 4b, right; see Supporting Information, Section 6). The destructive interference of emission due to *i*_*x*_ and *i*_*z*_ currents with the strong nonlinear contribution (Figure 4b, middle) therefore allows the shift currents to become the dominant mechanism for out-of-plane ribbons, producing the phase-shifted THz pulses. Interestingly, for very small ribbon sizes, the two current contributions are predicted to add constructively. Although we did not explore the emission from very small ribbons experimentally here, we note that smaller nanostructures may suppress these currents as a uniform distribution of carriers develops more quickly in a smaller volume, diminishing their contribution to the THz emission.

## Conclusions

In conclusion, we have demonstrated carrier-envelope phase control of THz emission from InAs metasurfaces by engineering the geometry and orientation of nanoscale ribbon arrays. The phase shift, Δϕ, spanning ±π/2, arises from the selective activation and interference of distinct THz generation mechanisms: lateral photocurrents, shift currents, and photocurrents normal to the surface (*i*_*z*_). This was achieved with a uniform linear polarization of the excitation beam (*s*-polarized). Numerical modeling reveals the interplay of these mechanisms and how ribbon size and orientation modulate their relative contributions, enabling constructive or destructive interference that governs both emission amplitude and phase—with in-plane ribbons dominated by lateral photocurrents and out-of-plane ribbons by shift currents. These results highlight how InAs metasurfaces, patterned into nanoscale ribbon arrays, can provide a versatile platform for THz wavefront synthesis. Integration of these ribbon arrays with demonstrated binary phase metasurfaces^30^ opens a pathway to THz pulse generation with arbitrary carrier-envelope phase, enabling structured THz wavefronts with tailored phase landscapes. This capability can lead to advancements in lightwave-driven scanning probe microscopy,^33,47^ subpicosecond control of electron dynamics^48^ and other nonlinear optics, imaging, and spectroscopy applications.

## Supplementary Material



## Notes

It contains additional information detailing sample fabrication and testing, experimental setup, and numerical simulations (PDF)
