# Supplementary material for: Carrier-Envelope Phase Control in Terahertz Pulse Generation Using InAs Ribbon Metasurfaces
Source: ACS Photonics. 2025 Jul 28;12(8):4534–9. doi: 10.1021/acsphotonics.5c00941 (PMC12372169; doi:10.1021/acsphotonics.5c00941)
Supplement: Supplementary file 1 [file ph5c00941_si_001.pdf]

Supporting Information

Carrier-envelope phase control in terahertz pulse generation using InAs ribbon metasurfaces

Sarah Norman[1], Hyunseung Jung[2,3], James Seddon[1], Samuel Prescott[1], C. Thomas Harris[2,3], Sadhvikas Addamane[2,3], Igal Brener[2,3] and Oleg Mitrofanov[1]

[1]Electronic and Electrical Engineering, University College London, Torrington Place, London, WC1E 7JE, UK

[2]Center for Integrated Nanotechnologies, Sandia National Laboratories, Albuquerque, New Mexico 87123, USA

[3]Sandia National Laboratories, Albuquerque, New Mexico 87123, USA

S1. InAs ribbon array fabrication

Ribbon array samples of different periods were fabricated from a uniform 130 nm thick indium arsenide (InAs) layer grown by molecular beam epitaxy (MBE) on a (100)-oriented GaAs substrate (wafer number VA1310). Prior to the InAs growth, two $Al_{0.55}Ga_{0.45}As$ stop-etch layers and a 100 nm GaAs spacer layer were grown at standard temperatures, while the InAs growth was initiated at 350 °C and gradually raised to ~450 °C.

Ribbon arrays were defined using electron-beam lithography with a dual-layer PMMA resist and a spin-coated HMDS adhesion layer. Then, a 400/20 Å SiO_2/Ti hard mask was deposited by e-beam evaporation, followed by pattern transfer to the InAs layer via inductively coupled plasma reactive ion etching (CH_4/H_2, 4 sccm each). The mask was removed in buffered oxide etchant. Arrays (*area* = 400×600 μm^2) of varying periods, ranging from P = 300–550 nm (fill factor ~0.6), and two orthogonal ribbon orientations were fabricated. SEM images of the different ribbon array sizes are shown in Fig. S1.

The samples were then bonded to a 0.5 mm thick sapphire substrate using ~2 μm thick EPO-TEK 353ND epoxy, with the structured surface facing the sapphire. The GaAs substrate and intermediate layers were removed by mechanical lapping and sequential wet etching, leaving only the InAs ribbon arrays atop the sapphire substrate.[1–3]

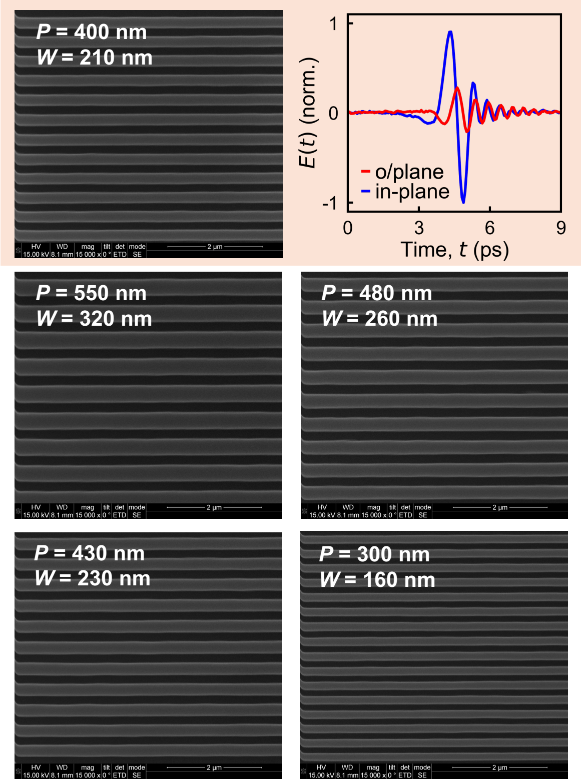

Figure S1. SEM images of InAs ribbon arrays with periods ranging from 300 nm to 550 nm (scale bar: 2 μm). The orange-shaded region highlights the ribbon array used in Figs. 1–4 in the Article, alongside time-domain waveforms of *p*-polarized THz emission from in-plane (blue) and out-of-plane (red) ribbons when illuminated with *s*-polarized optical excitation.

Two sets of ribbon arrays were fabricated on the same sample (set A and set B in Fig. S2) where ribbons were aligned along the [110] InAs crystal axis for set A and along the [$\bar{1}$10] crystal axis for set B. The data in Figs. 1, 3, and 4 were collected using the ribbon arrays with a 400 nm period from set A (in-plane ribbon orientation) and set B (out-of-plane ribbon orientation). The data in Fig. 2 shows THz emission from the 400 nm period ribbon array in set A as it was incrementally rotated from 0° to 90°.

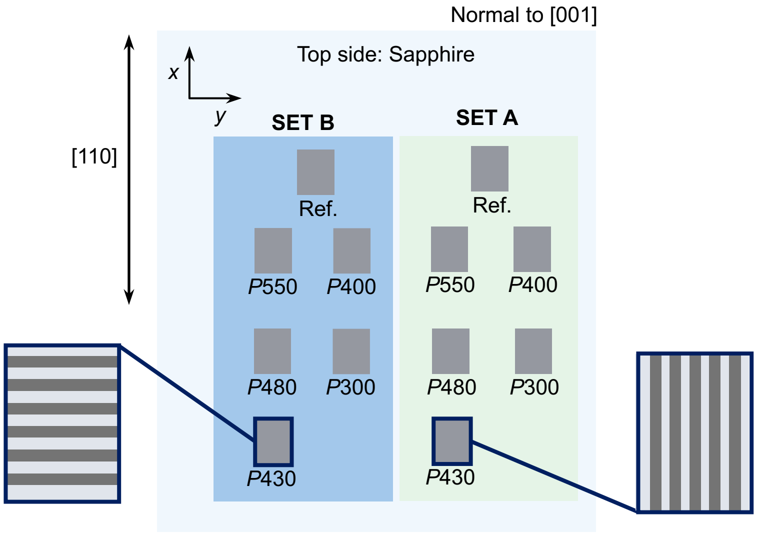

Figure S2. Schematic of the sample layout showing the two sets of InAs ribbon arrays (set A and set B) with periods ranging from 300–550 nm. Set A (containing ribbons aligned along the [110] crystal axis) was used for characterization of THz emission for the in-plane ribbon orientation, while set B (containing ribbons aligned along the [$\bar{1}$10] axis) was used for characterization of THz emission for the out-of-plane ribbon orientation.

S2. Experimental setup

THz pulse generation was characterized using a THz time-domain spectroscopy setup. The InAs ribbon arrays were excited by 100 fs pulses from a Ti:Sapphire laser ($\lambda = 800$ nm, average power ~500 mW, repetition rate 76 MHz). The experimental setup is illustrated in Fig. S3. A 0.650 mm thick (100) GaAs wafer was placed at the back of the sapphire substrate to block the optical excitation while transmitting the THz pulses. Time-domain waveforms of the emitted THz pulses were measured using a photoconductive antenna detector with a 10×10 μm^2 input aperture, positioned ~6 mm from the InAs ribbon arrays. The excitation beam was modulated at 1.7 kHz using a mechanical chopper, and the detector signal was pre-amplified and demodulated at the modulation frequency using a lock-in amplifier.

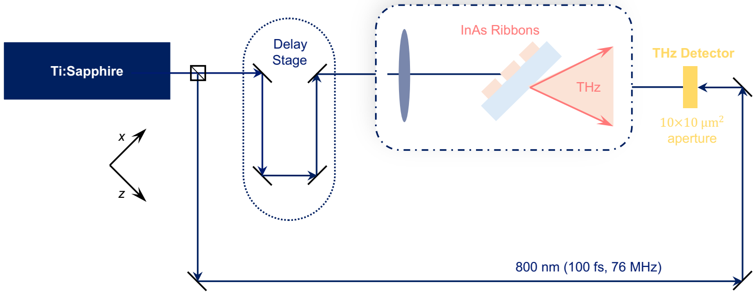

Figure S3. Schematic diagram of the experimental setup, where a convex lens creates an excitation spot of 160 ± 12 μm in diameter within the ribbon arrays, positioned slightly closer than the focal plane of the lens. The emitted forward-propagating THz pulses were detected with a photoconductive antenna detector.

The incident excitation was aligned to each array and characterized using a video camera (Thorlabs) (Fig. S4a). Due to saturation of the camera sensor at the beam centre, the intensity profile was clipped at the top (Fig. S4b, c); nevertheless, a Gaussian function could be fitted to the unsaturated regions of the profile to extract the full width at half maximum (FWHM). Averaged over several measurements in each experiment, the FWHM of the optical excitation beam was 160 ± 12 μm.

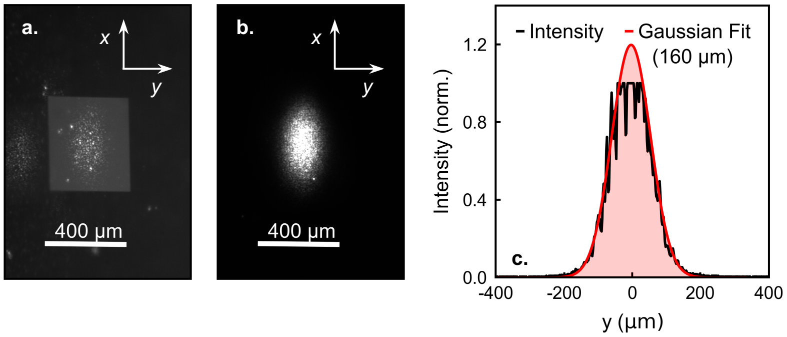

Figure S4. Images of the incident optical excitation beam centred on a ribbon array (a), and on an unpatterned region of the same sample (b). The beam appears elliptical in the image due to the sample being viewed approximately at a normal to the sample surface while the excitation beam is incident an angle of ~45°. (c) Intensity profile of the excitation beam from (b) fitted with a Gaussian function.

We also modeled the far-field emission pattern for a Gaussian beam of a diameter ranging from 150 μm to 300 μm (Fig. S5). The experimentally observed emission pattern is close to patterns expected for a

Gaussian-profile source with a FWHM between 150 and 200 μm, which is in excellent agreement with the size of the incident optical beam (FWHM = 160 ± 12 μm).

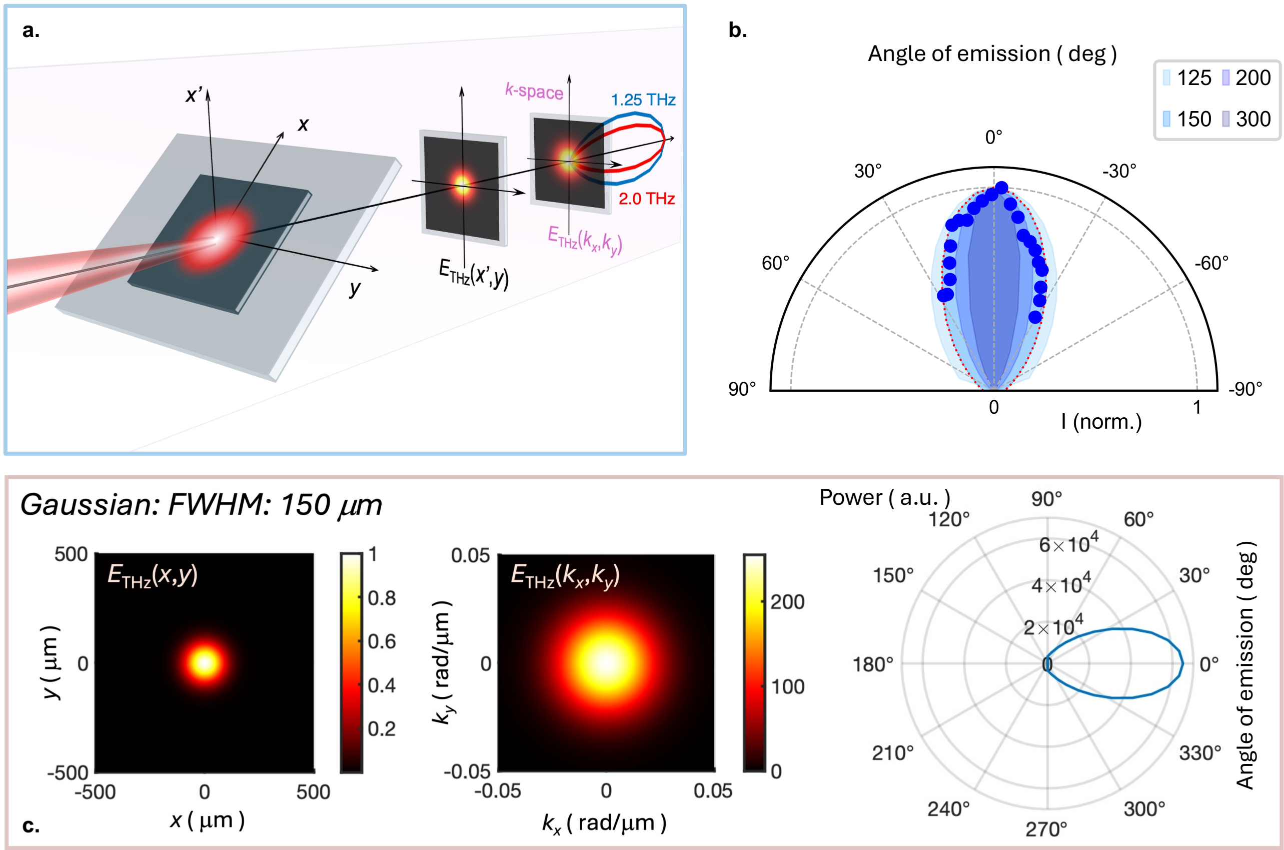

Figure S5. (a) Schematic of the experimental configuration illustrating the spatial distribution of generated THz waves in real space (*x'*,*y*) and reciprocal space (k_x,k_y), and the angular emission patterns for two THz frequencies. (b) Simulated angular emission profiles at 1.25 THz for Gaussian beams with diameters of 150 μm, 200 μm, and 300 μm. The red dashed line marks the emission pattern for the 150 μm Gaussian beam diameter (the closest modeled size to the measured spot with FWHM = 160 ± 12 μm). (c) Real-space spatial profiles of the excitation beam, corresponding distributions in reciprocal space (*k*-space) and resulting angular emission patterns derived from the k-space distribution

S3. Design of THz metasurface emitter for generation of THz pulses with arbitrary phase

Distinct THz generation mechanisms (shift currents and lateral photocurrents) can be selectively activated in the ribbon arrays with different orientations enabling control of the carrier-envelope phase between 0 and $\pm\pi/2$ (Article). In Fig. S6, we illustrate a metasurface for generation of THz pulses with intermediate

phase, for example, $\pi/4$. This metasurface comprises both in-plane and out-of-plane ribbon array segments, and the phase is determined by the ratio of segment areas (Fig. S6a,b). For the phase of $\pi/4$, the metasurface can be divided into subwavelength-size 'super cells,' each containing 16 segments: 4 in-plane and 12 out-of-plane ribbon array segments (a larger total area for the out-of-plane ribbon arrays compensates for the lower amplitude THz pulses produced). A modelled superposition of THz pulses generated from the 16 segments showing the expected phase of $\pi/4$ (Fig. S6c, black line).

The granularity of phase control in this approach is set by the minimal size of the ribbon array segments. For example, a 300×300 μm^2 super cell divided into 10×10 μm^2 segments could have 900 individual elements, resulting in fine increments of possible phase values (finer than one degree).

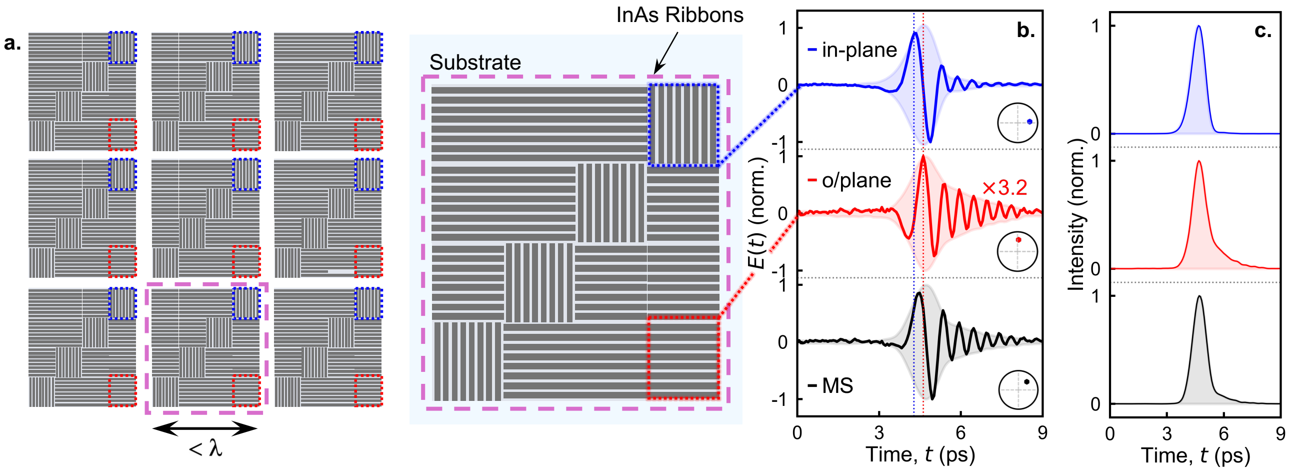

Figure S6. (a) Schematic of a metasurface (left) comprised of super cells which consists of 4 in-plane and 12 out-of-plane (o/plane) ribbon segments (right) to produce a carrier-envelope phase of $\phi = \sim\pi/4$. (b) A synthesised waveform (black line) expected from the metasurface with in-plane and out-of-plane ribbon segments, each generating THz pulses with discrete phases, 0 (blue) and $\pi/2$ (red) respectively (Article). Shaded regions indicate the amplitude envelope of each pulse. *Insets*: polar diagrams illustrating the corresponding phase at 1 THz (relative to the phase generated in a uniform InAs layer). (c) Intensity envelope for THz pulses in (b).

The full 0–2π phase coverage is theoretically possible. For example, InAs cubic resonator arrays offer a possibility of switching the polarity of generated THz pulses, equivalent to a π phase shift, by changing the incident excitation from *s*-polarized to *p*-polarized.[1,2] Desired polarization for each individual segment can be achieved with a spatial light modulator. Furthermore, the lateral photocurrent effect for the in-plane

ribbon arrays enables the polarity switching by simply changing the sample tilt from 45° to −45°. By combining the polarity switch (discrete π phase shift) with the continuous $\pm\pi/2$ phase tuning demonstrated in this study, THz pulses with arbitrary carrier-envelope phase, in principle, can be generated.

S4. THz pulse generation from InAs ribbon arrays of variable sizes

We investigated the dependence of THz emission on ribbon size by characterizing five metasurfaces with array periods ranging from 300 nm to 550 nm, using the experimental setup described in Section 2. The fill factor of ~0.6 remained constant across all arrays, such that an increase in ribbon width, W, was proportional to an increase in period, P. Peak THz pulse amplitudes are shown in Fig. 4 of the Article, while full time-domain waveforms and corresponding spectra are provided in Fig. S7.

While we did not directly measure the optical-to-THz conversion efficiency, we benchmarked the emission from the ribbon arrays against that of an unstructured InAs layer of equal thickness (Fig. 1b and Fig. S7). The ribbons with strongest emission (period P = 430 nm, width W = 230 nm) produce THz emission ~1.4× greater in amplitude than that of the uniform InAs. We therefore estimate that the optical-to-THz conversion efficiency for the InAs in-plane ribbon arrays is $\approx 1 \cdot 10^{-4}$ or (0.01 %).[1]

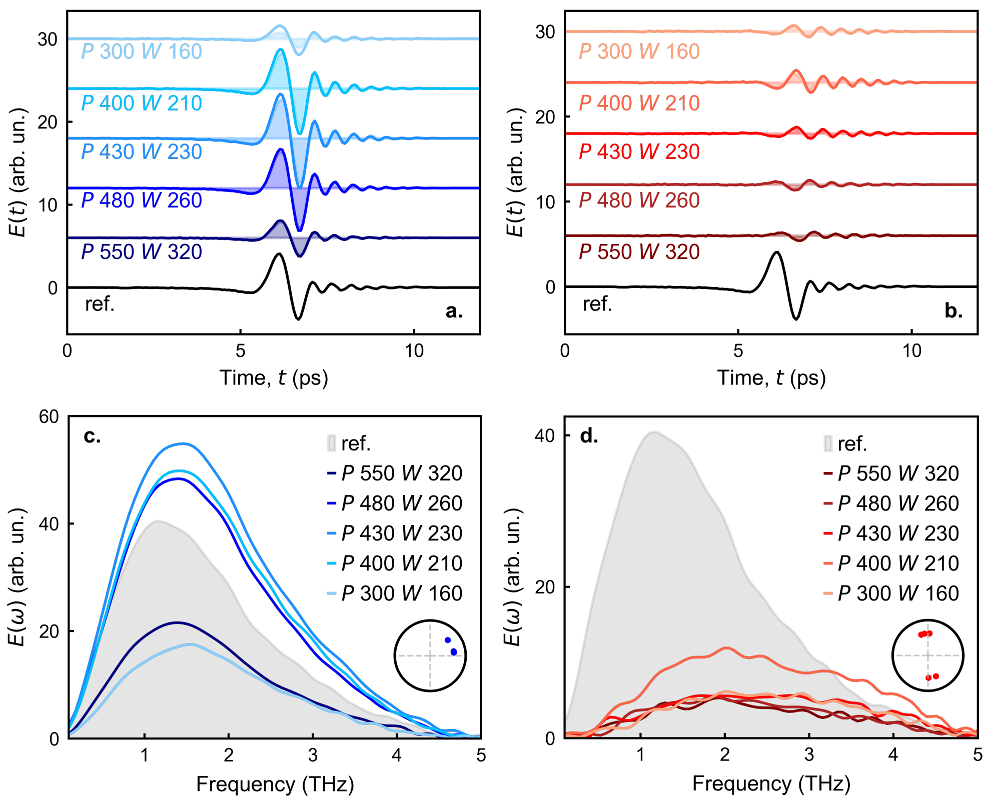

Figure S7. *p*-polarized THz pulses generated from (a) in-plane ribbon arrays (blue waveforms), patterned along the [110] (solid lines) and [$\bar{1}$10] (shaded regions) crystal axes, for various ribbon periods (300–550 nm). A reference pulse generated from a uniform InAs layer (with *p*-polarized incident excitation) is shown using a black line. (b) *p*-polarized THz pulses generated from out-of-plane ribbon arrays (red waveforms), patterned along the [$\bar{1}$10] (solid lines) and [110] (shaded regions) crystal axes. (c,d) Spectral amplitude of the (solid lines) generated pulses in (a,b). *Insets*: polar diagrams illustrating the phase of the emitted waves at 1 THz (relative to that for a uniform InAs layer).

S5. THz emission from lateral photocurrents

Lateral photocurrents arise from gradients in the photoexcited charge carrier density (lateral photo-Dember effect). They are formed when the semiconductor is photoexcited with spatially non-uniform ultrafast optical pulse[4] and/or when the pulse sweeps across the surface at an oblique angle (Fig. S8).[5,6] By modeling the photoexcitation process (100 fs, 100 μm spatial FWHM), we calculated the resulting charge carrier density gradient and corresponding lateral photocurrents. Then, we evaluated the emission of THz pulses from the temporal derivative of the lateral photocurrents (Equation 2, in S6).

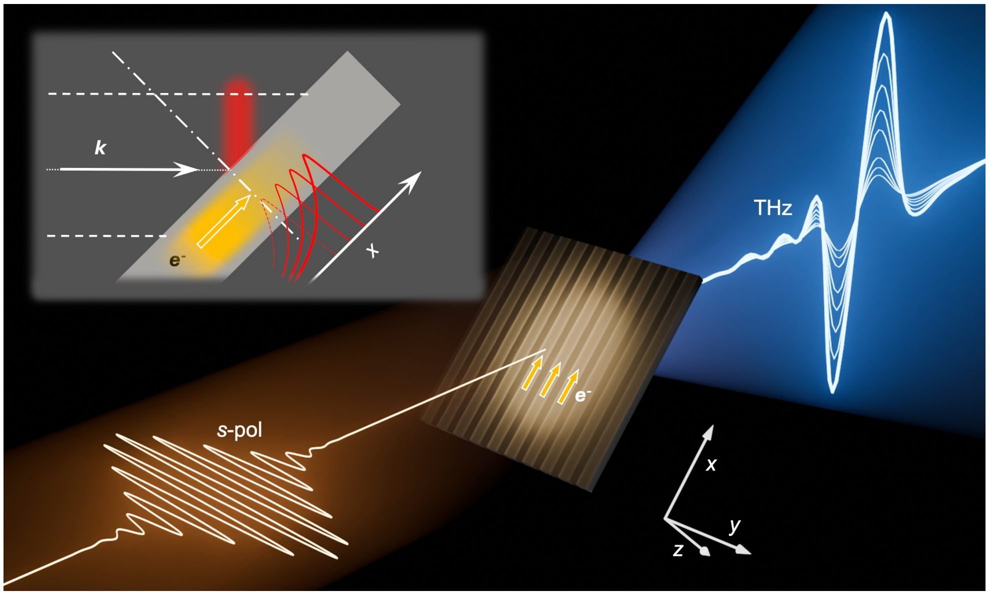

Figure S8. Illustration of THz emission due to lateral photocurrents along the ribbons (*x*-direction). InAs ribbons (grey) are illuminated with ultrafast optical pulses at a 45° incidence angle, generating charge carriers and driving THz emission. *Inset*: side-view schematic of the InAs ribbons showing the incident optical excitation (red), carrier motion (yellow), and the charge carrier density $n_e(x)$ along the surface as the excitation pulse sweeps along the ribbons.

Detailed results of this model are shown in Figs. S9: (a)-(d) illustrate the photoexcited charge carrier dynamics at normal incidence, where the excitation is symmetric and no net lateral photocurrents are generated. In contrast, (e)-(h) show the same quantities under 45° incidence, where a net lateral photocurrent and THz emission arise due to an asymmetric photocurrent distribution.

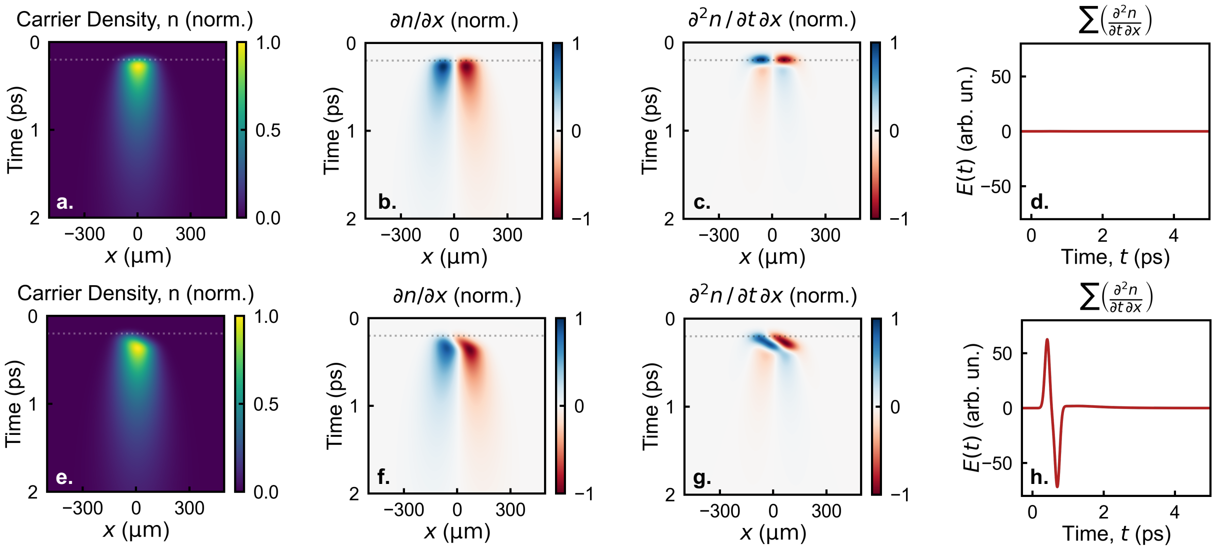

Figure S9. Modeling of photoexcited charge carrier dynamics and THz pulse generation at normal incidence. (a) Spatiotemporal evolution of charge carrier density $n_e(x,t)$. (b) Gradient of the carrier density ($\partial n_e/\partial x$) driving lateral photocurrents. (c) Temporal derivative of the lateral photocurrent ($\partial^2 n/\partial x\partial t$), which defines the waveform of the THz pulse, calculated as a sum of all current contributions over the sample area (d). (e–h) Corresponding plots for a 45° incidence angle, showing the same quantities as in (a–d).

S6. Numerical evaluation of THz generation in InAs metasurfaces

InAs is a widely used THz emitter, which supports several generation mechanisms, including the photo-Dember effect, optical rectification, shift currents, and lateral photocurrents,[2,5,7,8] with selected mechanisms predicted to exhibit a relative phase shift of $\pi/2$.[9] All these mechanisms scale with the pump intensity, which, in the case of excitation above the bandgap, determines the average density of charge carriers generated within the ribbon arrays. Nonlinear mechanisms such as shift currents and bulk optical rectification are also sensitive to the distribution of vectorial components of the excitation field.[2,3] To obtain the spatial distribution of the intensity and distributions of vectorial components of the electric field in the ribbons, we used a finite integration technique (CST Microwave Studio).

We considered the following distinct THz generation mechanisms: shift currents, lateral photocurrents, photocurrents due to carrier density gradients, e.g., a current normal to the surface (i_z), and optical rectification.

Shift currents:

This mechanism generates THz pulses with the electric field, $E_{shift}(t)$, proportional to the temporal derivative of the intensity evolution of the excitation pulse, $I(t)$:[10]

$$i_{shift}(t) \propto E(t)E^*(t) \propto I(t), \quad E_{shift}(t) \propto \frac{dI(t)}{dt}. \tag{1}$$

Lateral photocurrents:

THz emission from lateral photocurrents arises from charge carrier density gradients,[5,6] with the radiated THz field, $E_{lat}(t)$, proportional to the evolution of intensity of the excitation pulse, $I(t)$, and exhibits a phase shift, $\Delta\phi = \pm\pi/2$, relative to the THz emission from shift currents:

$$i_{lat}(t) \propto \frac{dn(t)}{dx}, \quad E_{lat}(t) \propto \frac{di_{lat}}{dt} \propto \frac{d}{dt}\left\{\frac{dn}{dx}\right\} \propto \frac{d}{dx}\left\{\frac{dn}{dt}\right\} \propto \sim \frac{d}{dx}I(t). \tag{2}$$

Charge-carrier density gradient currents:

THz emission arising from charge carrier density gradient currents within ribbons, specifically i_z, and in the case of out-of-plane ribbons, i_x, as well as drift currents due to built-in electric fields, is expected to follow the same temporal dependence as Eq. 2.

Optical rectification currents:

Finally, THz pulse generation due to bulk optical rectification, $E_{rect}(t)$, (non-resonant nonlinear process) is also present; however, it is expected to produce a significantly weaker contribution compared to the shift currents[9]

$$E_{rect}(t) \propto \frac{d^2P_{NL}}{dt^2} \propto \frac{d^2I(t)}{dt^2}. \tag{3}$$

In the table below, we summarize the expected contribution of these mechanisms to the THz generation from InAs ribbon arrays based on our numerical and analytical modeling, as well as the relevant experimental observations.

**THz Generation Mechanism**	**Expected Contribution** (in ribbon arrays)	**Temporal evolution** (model)	**Experimental Observations**
Lateral photocurrents, i_{lat}	**IpR:** activated<br>**OpR:** negligible	$E_{lat}(t) \propto \sim \frac{d}{dx} I(t)$	**IpR:** strongest emission except for small ribbon sizes (Figs. 4a, S7).<br>**OpR:** reduced emission compared to IpR.
Shift currents, i_{shift}	Activated, comparable for **IpR** and **OpR.**	$E_{shift}(t) \propto \frac{dI(t)}{dt}$	**OpR:** phase-shifted emission (Fig. 1)
Charge carrier gradients, i_{gr}	Defined by the intensity distribution inside the ribbons.	$E_{i_{gr}}(t) \propto \sim \nabla I(t)$	**IpR:** peak in THz emission for ribbons with a period of ~450 nm (Figs. 4a, S7).
Built-in field photocurrents, i_B	Negligible due to small band-bending in InAs[11].	$E_B(t) \propto \sim I(t)$	N/A
Optical rectification, i_{rect}	Significantly weaker contribution compared to shift currents.	$E_{rect}(t) \propto \frac{d^2 I(t)}{dt^2}$	N/A

Table Notations:

IpR – In-plane ribbons
OpR – Out-of-plane ribbons
E_{i_gr} – THz electric field generated by charge carrier gradients.
E_B – THz electric field generated from photocurrents driven by built-in electric fields

Electromagnetic Simulations:

The InAs ribbon arrays were modeled using the Finite Integration Technique (FIT) in CST Microwave Studio. The structure was simulated using a unit cell with periodic boundary conditions in the plane of the array and open boundaries along the top and bottom surfaces. Ribbon arrays with periods ranging from 200 nm to 600 nm were modeled, maintaining a consistent fill factor (~0.6). The excitation wave with a wavelength of 800 nm and an incidence angle of 45° was modeled using the Floquet port and the first Floquet port mode. From these simulations, we extracted the optical absorption within the ribbons and the spatial distribution of vectorial electric field components.

Variation of the THz emission from lateral photocurrents with the array period was calculated assuming that the THz field amplitude scales linearly with the optical absorption, which determines the total number

of charge carriers generated within the ribbons (Eq. 2). The variation of the THz emission from shift currents was calculated from the electric field components within the ribbons (Eq. 1) and the non-linear tensor.[2] To evaluate the variation of the THz emission due to the photoexcited charge carrier density gradients within the ribbons across different ribbon periods, we extracted the intensity distribution $I(x,y,z)$ within the ribbon volume from the simulations and then calculated the corresponding transient current vector components as the gradient of charge carrier density, $i_{gr}(x,y,z) \propto \sim\nabla I(x,y,x)$.

REFERENCES:

1. Jung, H. *et al.* InAs terahertz metalens emitter for focused terahertz beam generation. *Adv. Photonics Res.* **5**, 2400125 (2024).
2. Jung, H. *et al.* Terahertz Pulse Generation with Binary Phase Control in Nonlinear InAs Metasurface. *Nano Lett.* **22**, 9077–9083 (2022).
3. Hale, L. L. *et al.* Terahertz Pulse Generation from GaAs Metasurfaces. *ACS Photonics* **9**, 1136–1142 (2022).
4. Klatt, G. *et al.* Terahertz emission from lateral photo-Dember currents. *Opt. Express* **18**, 4939–4947 (2010).
5. Mueckstein, R. *et al.* Near-field analysis of terahertz pulse generation from photo-excited charge density gradients. *IEEE Trans. Terahertz Sci. Technol.* **5**, 260–267 (2015).
6. Corzo-Garcia, S. C., Hernandez-Serrano, A. I., Castro-Camus, E. & Mitrofanov, O. Monte Carlo simulation of near-field terahertz emission from semiconductors. *Phys. Rev. B.* **94**, 045301 (2016).
7. Gu, P., Tani, M., Kono, S., Sakai, K. & Zhang, X.-C. Study of terahertz radiation from InAs and InSb. *J. Appl. Phys.* **91**, 5533–5537 (2002).
8. Reid, M., Cravetchi, I. V. & Fedosejevs, R. Terahertz radiation and second-harmonic generation from InAs: Bulk versus surface electric-field-induced contributions. *Phys. Rev. B Condens. Matter* **72**, 035201 (2005).
9. Côté, D., Laman, N. & van Driel, H. M. Rectification and shift currents in GaAs. *Appl. Phys. Lett.* **80**, 905–907 (2002).
10. Tonouchi, M. Simplified formulas for the generation of terahertz waves from semiconductor surfaces excited with a femtosecond laser. *J. Appl. Phys.* **127**, 245703 (2020).
11. Johnston, M. B., Whittaker, D. M., Corchia, A., Davies, A. G. & Linfield, E. H. Simulation of terahertz generation at semiconductor surfaces. *Phys. Rev. B* **65**, 165301 (2002).
